# Variant Prediction by Analyzing RdRp/S Gene Double or Low Amplification Pattern in Allplex SARS-CoV-2 Assay

**DOI:** 10.3390/diagnostics11101854

**Published:** 2021-10-08

**Authors:** Min-Kyung So, Sholhui Park, Kyunghoon Lee, Soo-Kyung Kim, Hae-Sun Chung, Miae Lee

**Affiliations:** Department of Laboratory Medicine, Ewha Womans University College of Medicine, Seoul 03760, Korea; mkso79@gmail.com (M.-K.S.); solheepark@ewha.ac.kr (S.P.); kama.kyunghoon@gmail.com (K.L.); skkim1@ewha.ac.kr (S.-K.K.); sunny0521.chung@ewha.ac.kr (H.-S.C.)

**Keywords:** SARS-CoV-2, Allplex SARS-CoV-2 Assay, B.1.671.2, spike gene, amplification curve

## Abstract

The spread of delta variants (B.1.671.2) of severe acute respiratory syndrome coronavirus 2 (SARS-CoV-2) is a severe global threat. Multiplex real-time PCR is a common method for confirming SARS-CoV-2 infection, however, additional tests, such as whole genomic sequencing, are required to reveal the presence or type of viral mutation. Moreover, applying whole genomic sequencing to all SARS-CoV-2 positive samples is challenging due to time and cost constraints. Here, we report that the double or low amplification curve observed during RNA-dependent RNA polymerase (RdRp) gene/S gene amplification in the Allplex SARS-CoV-2 Assay is related to delta/alpha variants. We analyzed the RdRp/S gene amplification curve using 94 samples confirmed as SARS-CoV-2 infection by the Allplex SARS-CoV-2 Assay from January to August, 2021. These positive samples identified variant types using the Novaplex SARS-CoV-2 Variants I and IV Assays. Overall, 17 samples showing a double curve and 11 samples showing a low amplification pattern were associated with alpha-/delta-type strains with variants in the P681 region. The double or low curve shown in the RdRp gene amplification curve had 100% sensitivity and 100% specificity for diagnosing delta/alpha variants. During the SARS-CoV-2 virus diagnostic RT-PCR test using the Allplex SARS-CoV-2 Assay, we could consider the presence of delta/alpha variants in the samples with double or low amplification curve of the RdRp/S gene channel. This PCR amplification curve abnormality enables rapid and cost-effective variant type prediction during SARS-CoV-2 diagnostic testing in clinical laboratories.

## 1. Introduction

Viruses are constantly changing as they acquire mutations. Several variants of severe acute respiratory syndrome coronavirus 2 (SARS-CoV-2) have emerged worldwide since late 2020 [1]. Among them, the delta variant strain (B.1.617.2) is currently the predominant global variant of the virus, including in Korea [2,3]. The delta variant threatens the world with increased infectivity and faster spread than the initial SARS-CoV-2 strain, the COVID-19-causing virus [4]. The gold standard method for diagnosing infections by a specific variant is to sequence the whole or partial genome of the virus [5]. While the sequencing method provides accurate information regarding variants, it remains expensive and time-consuming, limiting its implementation in all diagnostic samples. Screening approaches based on PCR methods are relatively cheaper, and the results can be checked in a few hours. Therefore, PCR methods are more feasible [5,6,7].

The Allplex SARS-CoV-2 Assay (Seegene, Seoul, South Korea), considered a full-approval reagent by the KFDA, is a multiplex real-time (RT) PCR assay to confirm SARS-CoV-2 infection. The assay contains four target genes (the envelope (E) gene of Sarbecovirus, the RNA-dependent RNA polymerase (RdRp) gene, S gene encoding spike protein, and nucleocapsid (N) gene of SARS-CoV-2) in one tube—the S and RdRp genes, among the four target genes, were detected in the same fluorescence channel. The majority of mutations in SARS-CoV-2 occur in the S gene encoding the spike protein, the primary antigen, and the altered S protein plays a role in infectivity, disease severity, and host immunity [8]. Moreover, if genetic changes occur in the target region of the S gene of the Allplex SARS-CoV-2 Assay, it can affect the efficiency of SARS-CoV-2 detection. 

We found that the cycle threshold (Ct) value of the RdRp/S gene was significantly delayed compared to the maximum Ct values of the E and N genes in the PCR test of one SARS-CoV-2 positive patient. We confirmed that the amplification curve of the sample exhibited an abnormal sigmoid curve with a second curve (double curve pattern) in the RdRp/S gene amplification channel. Ibba et al. reported, for the first time, the amplification curve pattern deviating from normal in a SARS-CoV-2 PCR assay concerning the alpha mutation (B.1.1.7) [9]. Therefore, we re-analyzed the RdRp/S target amplification curve in SARS-CoV-2 positive samples confirmed by the Allplex SARS-CoV-2 Assay. This study aimed to determine the relationship between the double or low RdRp/S gene amplification curve and specific variant types in clinical specimens, and to investigate the clinical performance of the double or low amplification curve by demonstrating the variants in the samples with or without amplification curve changes.

## 2. Materials and Methods

### 2.1. Study Design

This study included 94 clinical respiratory specimens (nasopharyngeal swabs or sputum) that tested positive for SARS-CoV-2 by the Allplex SARS-CoV-2 Assay (Seegene, Seoul, South Korea) from January to August 2021 in a single hospital in South Korea. When there were multiple samples from the same patient in the same specimen type, a sample with a sufficient amount was randomly selected. The remaining samples were stored at −70 °C and thawed for variant type assays.

### 2.2. RNA Extraction and SARS-CoV-2 Detection Using Allplex SARS-CoV-2 Assay

The AdvanSure Nucleic Acid R or D Kits (LG Chem, Seoul, South Korea) platform was used to extract RNA from nasopharyngeal swabs in viral transport media or sputum according to the manufacturer’s instructions. 

The Allplex SARS-CoV-2 Assay, which targets four genes in a single tube (E, N, RdRp, and S genes), was used to test SARS-CoV-2 infection. PCR amplification was performed using a CFX-96 real-time thermocycler (Bio-Rad Laboratories Inc., CA, USA). Ct values were acquired from FAM (E gene), Quasar 670 (N gene), Cal Red 610 (RdRp and S genes), and HEX (internal control) channels. 

The RT-PCR products were considered positive if E, N, and RdRp/S targets were amplified with Ct ≤ 40. If only one or two gene targets amplified with Ct ≤ 40, the sample was considered inconclusive and was excluded from the positive specimens in this study. Following assay completion, target amplification curve analysis was performed using Seegene Viewer ver3.24 (Seegene, Seoul, South Korea).

### 2.3. Variant Type Detection

The same RNA extractions from the 94 positive samples were tested for variant type detection using a Novaplex SARS-CoV-2 Variants I Assay (Variants I Assay; Seegene, Seoul, South Korea) and a Novaplex SARS-CoV-2 Variants IV Assay (Variants IV assay; Seegene, Seoul, South Korea), following the manufacturer’s instructions. 

The Variants I Assay and Variants IV Assay include three target mutations in the S gene each, respectively: 69/70del; E484K; and N501Y for Variants I and K417N; L452R; and P681R for Variants IV. Additionally, the RdRp gene in the I assay was used to confirm the presence of SARS-CoV-2. Variant type determination was based on the combination of detected variants: alpha (B.1.1.7; 69/70del, N501Y); beta (B.1.351; E484K, N501Y, K417N); gamma (P.1; E484K, N501Y, K417T); delta (B.1.617.2; L452R, P681R); eta (B.1.525; 69/70del, E484K); and kappa (B.1.617.1; L452R, P681R; Supplementary Table S1). Only one mutation detection, such as E585K or E484K only, was considered indeterminate. If the amplification of the RdRp gene was undetected in the Variants I Assay, it was considered invalid. 

### 2.4. Data Analysis

Statistical analysis of the sensitivity or specificity and a t-test for comparing Ct values were done with MedCalc Statistical Software version 20.013 (MedCalc Software Ltd, Ostend, Belgium). For the sensitivity and specificity analysis of the Allplex SARS-CoV-2 RdRp/S double or low amplification curve pattern, delta or alpha variant samples identified by the variant detection kits were regarded as true positive samples. Samples with other variant types, indeterminate, or with no variant were considered true negative samples. We excluded invalid samples for the sensitivity and specificity calculation.

## 3. Results

**Figure 1:**
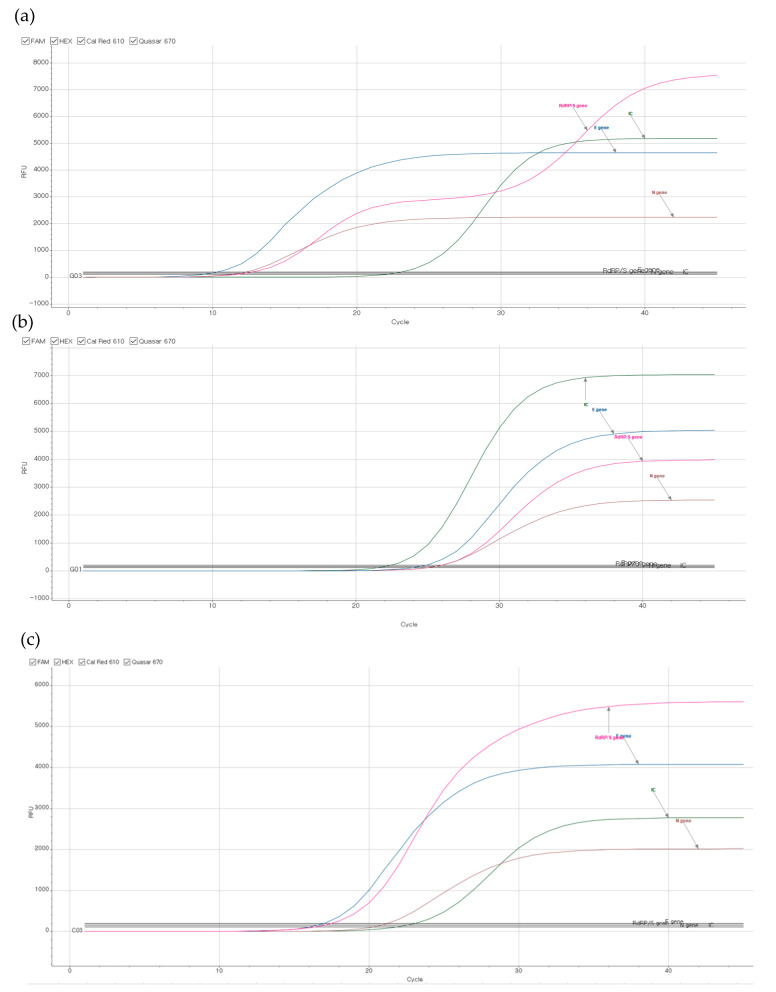
Representative RT-PCR amplification curve results; (**a**,**b**) COVID-19 positive samples revealed with delta variant with (**a**) a double curve pattern of RdRp/S, or (**b**) a single curve pattern and lower fluorescence units of RdRp/S than E gene and N gene. (**c**) COVID-19 positive samples with no specific variant: RdRp/S curve shows single amplification pattern with higher fluorescence units than E gene. Pink color: RdRp/S gene, blue color: E gene, brown color: N gene, green color: internal control.

Ninety-four SARS-CoV-2 positive samples were analyzed for the RdRp/S gene amplification curve of SARS-CoV-2 using Seegene Viewer ver3.24. We noticed three kinds of RdRp/S amplification patterns (Figure 1). 

In 17/94 samples, we found a double amplification curve pattern in the RdRp/S target gene channel. In 11 samples, each RdRp/S amplification curve was a single amplification curve (sigmoidal shape), but we noticed that the relative fluorescence units (RFU) of the RdRp gene were lower than others. The saturation curve of the RdRp/S gene was placed between the E gene and N gene. The remaining 66 samples resulted in the single RdRp/S amplification curves placed above the E gene curve. The representative samples showing a double curve pattern and a single curve with low amplification in the RdRp/S gene channel are presented in Figure 1a,b. Figure 1c represents a sample with no variant detection showing a single RdRp/S curve with high amplification RFU. 

By testing the variant types of 17 cases with the double curve pattern, L452R and P681R were simultaneously detected in 16 samples, and identified as delta variants (Table 1). In one sample, 69/70del and N501Y were positive, indicating an alpha variant. All 11 cases showing the single curve with low amplification pattern in the RdRp/S gene channel also revealed the delta variant by simultaneously detecting L452R and P681R mutation. Their RdRp/S gene amplification Ct values were 28.38 ± 4.06 (mean ± standard deviation, SD), which showed a statistically significant delay compared to the Ct values of 17.60 ± 4.40 (mean ± SD, p value < 0.0001) in the samples with the double curve pattern.

In 66/94 samples showing a single curve with high amplification pattern in the RdRp/S gene channel (Figure 1c), there was no alpha or delta variant. Among the 66 samples, 37 samples did not detect any mutation. Two samples were eta variant strains that detected 69/70del and E484K mutations (Table 1). In addition, in 25 samples, only one mutation was observed and was thus determined to be indeterminate (24 E484K or 1 L452R only; Table 1). The mutation detection results in each sample are presented in Supplementary Table S2. Two samples were considered invalid due to the absence of RdRp gene amplification in the Novaplex SARS-CoV-2 Variants I Assay. The Ct values of the RdRp/S gene in the two invalid samples by Allplex SARS-CoV-2 Assay were 38.22 and 34.93. 

When analyzing the distribution pattern of variant types of SARS-CoV-2 positive samples by month, no delta variant (0%, 0/48) was found from January to May, 2021. Alternatively, it was confirmed that the delta variant changed to the dominant species with 33% (4/12) in June, 60% (16/27) in July, and 100% (7/7) in August. 

We analyzed the diagnostic sensitivity and specificity of whether the double or low amplification curve pattern of the RdRp/S gene could diagnose SARS-CoV-2 delta or alpha variant strains. We obtained 100.0% sensitivity (95% CI: 87.7–100.0), and 100.0% specificity (95% CI: 94.4–100.0; Table 2). Regardless of the prevalence, the positive predictive value and negative predictive value were both 100.0%.

## 4. Discussion

The Allplex SARS-CoV-2 Assay is a SARS-CoV-2 detection kit commonly used in clinical laboratories for COVID-19 diagnosis. The principal of this kit, which comprises four fluorescence channels, is real-time multiplex PCR to detect four target genes (E gene of Sarbecovirus, and the N, RdRp, and S gene for SARS-CoV-2). Among the four target genes, the RdRp and S genes were designed to be detected in one channel by using the similarity in amplification efficiency. When a variant occurs at the target site, the amplification efficiency of the target gene may decrease, and amplification of the mutated gene may start later than other genes, causing no curve or a delayed curve. The S protein of SARS-CoV-2 is composed of S1 and S2 subunits, and there is a furin cleavage site at the interface between the two units to facilitate transmission [10]. When the P681 site of SARS-CoV-2 is mutated, viral replication changes as the cleavage site is altered [11]. The delta variant strain includes a mutation in which the amino acid proline at position 681 changes to arginine and that of the alpha variant strain changes to histidine [12]. 

Our clinical laboratory has detected a double or low amplification curve pattern of the RdRp/S gene channel in several COVID-19 positive samples since June 2021. As a result of the retrospective analysis of 94 PCR amplification curves positive for SARS-CoV-2 from January to August 2021, 28 samples that showed a double or a low amplification curve in the RdRp/S gene channel were identified as delta or alpha variants (Table 1). Based on these results, although it was difficult to distinguish between alpha and delta variants with the RdRp/S curved pattern, we can consider that the virus showing the abnormal curve in the RdRp/S gene channel has a mutation in the P681 position of the S gene. When a change occurs around the P681 region in the S gene, the target’s amplification efficiency decreases compared to the RdRp target gene, and then a double curve can be shown in the Allplex SARS-CoV-2 Assay. 

While all 17 samples showing double curves were delta or alpha variants, eleven delta variants showed single curves without double curves. We can consider two possibilities for the low amplification curve. One could be due to low viral load. It was confirmed that the Ct value (mean ± SD; 17.60 ± 4.40) in the RdRp/S gene channel of the samples showing the double curve was statistically significantly lower than the Ct value (mean ± SD; 28.38 ± 4.06) of the samples showing the single curve and the variant. The reason for this phenomenon is that the sample showing a single curve has a low viral load, therefore, it is considered that the amplification reaction was terminated before the second curve was generated. Another explanation could be dropout impact due to target partial mismatch [13,14]. This could fail S gene targeting and preserve only the PCR reaction in the RdRp gene. Therefore, the amplification level in a RdRp/S gene channel could drop compared to samples with non-P681 mutation. 

The delta variant, first discovered in India in October 2020, is currently spreading worldwide because of its high contagiousness, posing a health threat related to SARS-CoV-2 infection. The predominance of the delta variant presents difficulties in preventing SARS-CoV-2 for the following reasons: enhanced transmissibility; the possibility of decreased vaccine effectiveness; and increased hospitalization [1,4]. According to the Korea Disease Control and Prevention Agency, delta variants were detected in 3.3% of Korea’s domestic SARS-CoV-2 infections reported in June 2021. In August 2021, which was the end of the study period, 90–94.5% of SARS-CoV-2 positive patients were confirmed to be infected with the delta variant, exhibiting a sharp increase [3,15]. Our laboratory noticed the abnormality of the RdRp/S amplification curve at the beginning of June 2021, strongly suggesting that the spread of the delta variant started in this region from at least June, 2021. 

Two variant detection kits, Variants I and IV, were used for variant type determination in this study. The Variants I Assay kit alone can detect alpha, beta, and gamma variants classified as Variant of Concern by the World Health Organization using three key mutations, namely 69/70del, E484K, and N501Y [16]. The Variants IV Assay alone can identify delta and delta plus mutations by detecting K417N, L452R, and P681R. By integrating the variant targets detected in these two kits, various mutation types could be predicted (Supplementary Table S1). These variant detection kits use the same RT-PCR method applied to the Allplex SARS-CoV-2 Assay, therefore, they can be effectively introduced in clinical laboratories that utilize the PCR method for SARS-CoV-2 assays. However, when confirming the delta variant with these two kits, it should be noted that these tests cannot differentiate between delta and kappa (B.1.617.1) variants. We detected the mutations simultaneously found in the delta and kappa variants, therefore, this study could not distinguish the kappa variant from the samples identified as the delta variant. Since the majority of the SARS-CoV-2 viruses currently prevalent in Korea are delta variants, it was considered that the virus strain demonstrated in this study could be regarded as a delta variant.

## 5. Conclusions

We observed abnormal (double or low amplification) curve patterns of the RdRp/S gene when SARS-CoV-2 was detected using the Allplex SARS-CoV-2 Assay. Using two variant detection kits, we confirmed that this abnormal curve could correlate with delta/alpha variants in clinical specimens. Considering that the Allplex SARS-CoV-2 kit is used to diagnose COVID-19 worldwide, this study could be helpful in predicting the presence of delta or alpha variants with cost-effective, simple, and rapid methods, sooner than additional tests, such as genome sequencing.

## Figures and Tables

**Table 1:** RdRp/S curve pattern and variant types of SARS-CoV-2 positive clinical samples using Allplex SARS-CoV-2 Assay.

	Detected Mutation and Variant Type							
	69/70del & N501Y	L452R & P681R	69/70del & E484K	E484K Only	L452R Only	None		
RdRp/S Curve Pattern	Alpha	Delta or Kappa	Eta	Indeterminate	Indeterminate	No Variant	Invalid	Total
Double curve	1	16	0	0	0	0	0	17
Single curve with low amplification	0	11	0	0	0	0	0	11
Single curve with high amplification	0	0	2	24	1	37	2	66
Total	1	27	2	24	1	37	2	94

**Table 2:** The sensitivity and specificity of RdRp/S double or low amplification curve pattern analysis.

		Variant type		
		Delta or Alpha	Others *	Total
Curve Pattern	Abnormal (Double + Single with Low amplification)	28	0	28
	Single with high amplification	0	64	64
	Total	27	64	92
Sensitivity	100.0%	(95% CI: 87.7–100.0)		
Specificity	100.0%	(95% CI: 94.4–100.0)		

*: included samples with eta, indeterminate, and no variant

## Data Availability

Data of this study can be available on request.

## Supplementary Materials

The following are available online at https://www.mdpi.com/article/10.3390/diagnostics11101854/s1, Table S1: Variant type determination using combined mutations, Table S2: SARS-CoV-2 positive sample information and variant determinate results by Novaplex SARS-CoV-2 Variants I Assay and IV Assay.
