# Supplementary material for: Variant Prediction by Analyzing RdRp/S Gene Double or Low Amplification Pattern in Allplex SARS-CoV-2 Assay
Source: Diagnostics (Basel). 2021 Oct 8;11(10):1854. doi: 10.3390/diagnostics11101854 (PMC8534604; doi:10.3390/diagnostics11101854)
Supplement: Supplementary file 1 [file diagnostics-11-01854-s001.zip › diagnostics-1409537-supplementary.pdf]

Table S1: Variant type determination using combined mutations

		Variants I Assay			Variants IV Assay		
	**Variant type (WHO label/name)**	**69/70del**	**E484K**	**N501Y**	**K417N**	**L452R**	**P681R**
WHO VOC (Variant Of Concern)	Alpha, B.1.1.7	∨		∨			
	Beta, B.1.351		∨	∨	∨		
	Gamma, P.1		∨	∨			
	Delta, B.1.617.2					∨	∨
	Delta plus, AY.1&2				∨	∨	∨
WHO VOI (Variant Of Interest)	Eta, B.1.525	∨	∨				
	Iota, B.1.526		∨				
	Kappa, B.1.617.1					∨	∨

Table S2. SARS-CoV-2 positive sample information and variant determinate results by Novaplex SARS-CoV-2 Variants I Assay and IV Assay.

**Sample Information**			**Allplex SARS-CoV-2 Assay**				**Novaplex SARS-CoV-2 Variants I Assay**					**Novaplex SARS-CoV-2 Variants IV Assay**				**Variant Type**
**Sample_ Number**	**Collection Date**	**Specimen**	**E gene (Ct.)**	**RdRp/S gene (Ct.)**	**N gene (Ct.)**	**RdRp/S Curve pattern**	**RdRP gene (Ct.)**	**E484K (Ct.)**	**N501Y (Ct.)**	**69/70 del (Ct.)**	**Internal control (Ct.)**	**L452R (Ct.)**	**P681R (Ct.)**	**K417N (Ct.)**	**Internal control (Ct.)**	
S1	2021-01-20	Nasopharyngeal	27.07	27.47	28.62	Single_high	29.45	-	-	-	23.9	-	-	-	23.85	No variant
S2	2021-01-20	Sputum	32.58	32.09	32.54	Single_high	35.49	-	-	-	23.56	-	-	-	23.63	No variant
S3	2021-01-22	Nasopharyngeal	26.52	26.73	26.93	Single_high	28.73	-	-	-	23.91	-	-	-	23.74	No variant
S4	2021-01-25	Nasopharyngeal	34.52	34.81	35.57	Single_high	37.51	-	-	-	24.95	-	-	-	24.98	No variant
S5	2021-01-28	Nasopharyngeal	35.87	35.38	37.27	Single_high	40.05	-	-	-	24.81	-	-	-	24.94	No variant
S6	2021-02-01	Nasopharyngeal	38	38.22	37.04	Single_high	-	-	-	-	27.08	-	-	-	27.24	Invalid
S7	2021-02-02	Nasopharyngeal	36.45	34.65	35.09	Single_high	37.61	-	-	-	25.95	-	-	-	25.98	No variant
S8	2021-02-08	Nasopharyngeal	33.51	33.95	34.28	Single_high	36.51	-	-	-	24.25	-	-	-	24.31	No variant
S9	2021-02-08	Nasopharyngeal	24.81	24.22	25.64	Single_high	26.26	-	-	-	25.48	-	-	-	25.24	No variant
S10	2021-02-08	Sputum	35.16	34.2	36.87	Single_high	37.81	-	-	-	26.02	-	-	-	26.3	No variant
S11	2021-02-09	Nasopharyngeal	22.08	21.7	24.11	Single_high	23.56	-	-	-	24.11	-	-	-	23.98	No variant
S12	2021-02-09	Sputum	19.92	19.46	21.22	Single_high	21.81	-	-	-	26.55	-	-	-	26.55	No variant
S13	2021-02-15	Nasopharyngeal	24.59	24.13	23.96	Single_high	26.76	-	-	-	23.5	-	-	-	23.79	No variant
S14	2021-02-15	Sputum	25.07	26.02	24.49	Single_high	27.53	-	-	-	23.7	-	-	-	23.65	No variant
S15	2021-02-15	Nasopharyngeal	29.33	29.68	29.93	Single_high	31.16	-	-	-	27.71	-	-	-	27.74	No variant
S16	2021-02-17	Nasopharyngeal	35.52	36.28	38.5	Single_high	39.42	-	-	-	24.43	-	-	-	24.34	No variant
S17	2021-02-17	Sputum	25.35	25.22	26.18	Single_high	26.88	-	-	-	25.39	-	-	-	25.59	No variant
S18	2021-02-19	Nasopharyngeal	26.03	25.74	26.33	Single_high	27.32	-	-	-	27.66	-	-	-	27.6	No variant
S19	2021-02-20	Sputum	35.9	34.6	35.51	Single_high	39.12	-	-	-	26.2	-	-	-	26.34	No variant
S20	2021-02-22	Nasopharyngeal	20.02	20.1	21.63	Single_high	21.96	-	-	-	26.39	-	-	-	26.58	No variant
S21	2021-02-22	Nasopharyngeal	22.11	22.01	23.48	Single_high	24.12	-	-	-	25.07	-	-	-	25.32	No variant
S22	2021-02-24	Nasopharyngeal	16.46	16.46	18.24	Single_high	18.04	-	-	-	23.31	-	-	-	23.82	No variant
S23	2021-02-25	Sputum	22	21.84	23.63	Single_high	23.37	-	-	-	25.45	-	-	-	25.27	No variant
S24	2021-02-25	Nasopharyngeal	23.07	22.87	23.48	Single_high	25.24	-	-	-	26.07	-	-	-	26.64	No variant
S25	2021-02-25	Nasopharyngeal	35.3	37.6	37.71	Single_high	39.67	-	-	-	26.38	-	-	-	26.59	No variant

S26	2021-02-26	Sputum	34.06	33.97	34.29	Single_high	38.03	-	-	-	26.4	-	-	-	26.49	No variant
S27	2021-03-04	Nasopharyngeal	26.95	27.32	27.91	Single_high	29.22	-	-	-	24.38	-	-	-	24.45	No variant
S28	2021-03-04	Nasopharyngeal	31.71	31.75	31.82	Single_high	33.53	-	-	-	25.72	-	-	-	25.76	No variant
S29	2021-03-10	Nasopharyngeal	30.77	31.21	31	Single_high	33.03	-	-	-	28.1	-	-	-	28.16	No variant
S30	2021-03-17	Nasopharyngeal	34.7	33.86	34.06	Single_high	37.43	-	-	-	24.98	-	-	-	25.13	No variant
S31	2021-03-26	Nasopharyngeal	28.3	28.39	29.99	Single_high	27.18	-	-	-	22.23	-	-	-	22.11	No variant
S32	2021-03-26	Nasopharyngeal	32.3	32.81	33.24	Single_high	32.9	-	-	-	21.82	-	-	-	21.98	No variant
S33	2021-03-31	Nasopharyngeal	10.97	12.05	12.47	Single_high	10.23	-	-	-	23.05	-	-	-	23.02	No variant
S34	2021-04-01	Nasopharyngeal	31.57	31.7	30.91	Single_high	32.06	-	-	-	21.12	-	-	-	21.18	No variant
S35	2021-04-06	Nasopharyngeal	17.12	16.53	19.77	Single_high	16.4	18.88	-	-	23.76	-	-	-	23.87	Indeterminate
S36	2021-04-06	Nasopharyngeal	29.82	29.5	29.18	Single_high	28.62	-	-	-	23.69	-	-	-	23.64	No variant
S37	2021-04-13	Nasopharyngeal	16.31	17.02	17.46	Single_high	15.41	-	-	-	21.65	-	-	-	21.79	No variant
S38	2021-04-27	Nasopharyngeal	18	17.68	22.84	Single_high	16.82	19.54	-	-	23.82	-	-	-	23.93	Indeterminate
S39	2021-04-28	Nasopharyngeal	24.71	25.19	25.34	Single_high	24.27	-	-	-	23.48	-	-	-	23.35	No variant
S40	2021-05-03	Nasopharyngeal	11.25	12.73	16.47	Single_high	10.91	-	-	-	24.33	14.27	-	-	24.3	Indeterminate
S41	2021-05-04	Nasopharyngeal	21.8	21.73	25.15	Single_high	21.7	23.73	-	-	25.67	-	-	-	25.51	Indeterminate
S42	2021-05-10	Nasopharyngeal	15.35	17.79	16.57	Single_high	14.98	17.29	-	15.31	21.34	-	-	-	21.81	Eta
S43	2021-05-10	Nasopharyngeal	13.38	16.4	15.93	Single_high	13.99	16.16	-	13.28	20.78	-	-	-	21.06	Eta
S44	2021-05-10	Nasopharyngeal	17.03	17.46	20.27	Single_high	17.14	19.07	-	-	23.34	-	-	-	23.49	Indeterminate
S45	2021-05-11	Nasopharyngeal	19.16	19.21	22.73	Single_high	18.83	21.09	-	-	22.69	-	-	-	23.01	Indeterminate
S46	2021-05-17	Nasopharyngeal	26.7	27.48	31.48	Single_high	28.02	29.76	-	-	22.61	-	-	-	22.74	Indeterminate
S47	2021-05-18	Nasopharyngeal	18.94	19.57	23.94	Single_high	18.69	20.37	-	-	21.57	-	-	-	21.63	Indeterminate
S48	2021-05-31	Nasopharyngeal	28.35	28.99	32.16	Single_high	28.18	31.12	-	-	20.3	-	-	-	20.25	Indeterminate
S49	2021-06-01	Sputum	27.33	27.55	31.48	Single_high	27.09	29.4	-	-	21.9	-	-	-	22.07	Indeterminate
S50	2021-06-10	Nasopharyngeal	26.45	26.9	31.59	Single_high	25.88	28.51	-	-	19.66	-	-	-	19.82	Indeterminate
S51	2021-06-11	Nasopharyngeal	10.42	12.36	11.42	Double	9.86	-	-	-	21.36	13.87	13.01	-	21.31	Delta
S52	2021-06-11	Nasopharyngeal	9.66	12.56	16.3	Single_high	12.5	16.15	-	-	22.82	-	-	-	22.81	Indeterminate
S53	2021-06-11	Nasopharyngeal	15.41	14.98	19.33	Single_high	16.15	18.51	-	-	23.16	-	-	-	23.34	Indeterminate
S54	2021-06-15	Sputum	23.11	23.76	23.57	Double	22.09	-	-	-	24.14	25.11	23.44	-	24.18	Delta
S55	2021-06-16	Nasopharyngeal	26.92	28.2	28.19	Double	26.56	-	28.98	26.63	22.68	-	-	-	22.6	Alpha

S56	2021-06-21	Nasopharyngeal	15.26	16.15	15.07	Double	14.84	-	-	-	23.77	17.72	16.1	-	23.88	Delta
S57	2021-06-21	Sputum	11.71	14.25	13.05	Double	13.95	-	-	-	23.82	16.26	14.75	-	23.93	Delta
S58	2021-06-22	Nasopharyngeal	26	25.8	30.36	Single_high	26.44	29.1	-	-	21.33	-	-	-	21.3	Indeterminate
S59	2021-06-28	Nasopharyngeal	24.58	24.02	28.67	Single_high	23.71	26.33	-	-	23.82	-	-	-	23.75	Indeterminate
S60	2021-06-29	Nasopharyngeal	24.79	24.59	29.23	Single_high	23.83	26.29	-	-	20.79	-	-	-	20.9	Indeterminate
S61	2021-07-02	Nasopharyngeal	9.68	11.79	10.66	Double	9.05	-	-	-	23.78	13.09	10.51	-	23.89	Delta
S62	2021-07-02	Nasopharyngeal	28.24	29.97	29.71	Single_low	27.76	-	-	-	22.61	31.29	28.8	-	22.59	Delta
S63	2021-07-05	Nasopharyngeal	10.36	12.73	11.72	Double	9.33	-	-	-	22.05	13.28	10.33	-	22.08	Delta
S64	2021-07-05	Nasopharyngeal	34.82	36.43	33.63	Single_low	33.89	-	-	-	23.72	37.06	34.47	-	23.91	Delta
S65	2021-07-06	Nasopharyngeal	10.27	10.04	15.7	Single_high	10.07	11.12	-	-	22.08	-	-	-	22.33	Indeterminate
S66	2021-07-06	Nasopharyngeal	30.54	32.39	29.65	Single_low	31.82	-	-	-	22.93	34.68	31.42	-	23.08	Delta
S67	2021-07-07	Nasopharyngeal	25.23	24.62	29.63	Single_high	25.48	27.08	-	-	23.05	-	-	-	22.97	Indeterminate
S68	2021-07-07	Nasopharyngeal	35.63	34.93	39.19	Single_high	-	-	-	-	23.9	-	-	-	22.89	Invalid
S69	2021-07-12	Nasopharyngeal	25.97	27.46	26.46	Single_low	24.97	-	-	-	23.67	28.54	26.33	-	23.33	Delta
S70	2021-07-12	Nasopharyngeal	24.13	23.71	28.51	Single_high	23.33	25.95	-	-	21.52	-	-	-	21.54	Indeterminate
S71	2021-07-14	Nasopharyngeal	18.47	19.26	18.58	Double	18.07	-	-	-	23.14	21.69	19.27	-	23.27	Delta
S72	2021-07-14	Nasopharyngeal	11.42	12.69	18.23	Single_high	14.06	16.05	-	-	22.42	-	-	-	22.84	Indeterminate
S73	2021-07-14	Nasopharyngeal	25.02	24.1	29.77	Single_high	23.97	27.18	-	-	22.44	-	-	-	22.54	Indeterminate
S74	2021-07-14	Sputum	17.07	16.57	21.8	Single_high	17.24	19.09	-	-	17.65	-	-	-	17.91	Indeterminate
S75	2021-07-15	Nasopharyngeal	15.86	18.43	15.63	Double	16.41	-	-	-	23.27	18.83	16.83	-	23.67	Delta
S76	2021-07-15	Sputum	15.27	16.52	15.99	Double	16.55	-	-	-	20.16	18.79	16.21	-	20.14	Delta
S77	2021-07-17	Sputum	14.95	15.34	15.18	Double	15.67	-	-	-	23.57	18.11	16.4	-	23.87	Delta
S78	2021-07-19	Nasopharyngeal	26.48	25.51	30.66	Single_high	26.31	29.16	-	-	22.85	-	-	-	22.86	Indeterminate
S79	2021-07-19	Sputum	25.64	24.77	30.01	Single_high	25.56	27.49	-	-	21.58	-	-	-	21.76	Indeterminate
S80	2021-07-19	Nasopharyngeal	22.87	24.32	24.24	Single_low	23.49	-	-	-	20.63	27.78	24.21	-	20.98	Delta
S81	2021-07-20	Sputum	20.37	21.79	20.63	Double	23.84	-	-	-	19.22	24.43	21.55	-	19.61	Delta
S82	2021-07-21	Nasopharyngeal	36.76	36.5	33.73	Single_high	35.55	-	-	-	24.55	-	-	-	24.59	No variant
S83	2021-07-21	Sputum	27.7	29.43	27.59	Single_low	25.82	-	-	-	23	29.15	26.68	-	23.13	Delta
S84	2021-07-21	Nasopharyngeal	26.13	27.03	26.06	Single_low	28.94	-	-	-	19.97	31	28.39	-	19.93	Delta
S85	2021-07-25	Nasopharyngeal	29.56	29.11	33.05	Single_high	29.95	32.76	-	-	22.52	-	-	-	22.59	Indeterminate

S86	2021-07-26	Sputum	23.28	23.68	23.1	Single_low	22.79	-	-	-	24.98	25.64	23.99	-	25.18	Delta
S87	2021-07-28	Nasopharyngeal	15.7	16.39	15.73	Double	14.88	-	-	-	22.48	18.82	16.41	-	22.68	Delta
S88	2021-08-02	Nasopharyngeal	21.7	22.1	21.61	Double	33.76	-	-	-	19.26	35.7	33.76	-	19.16	Delta
S89	2021-08-04	Nasopharyngeal	16.77	16.94	15.39	Double	16.64	-	-	-	24.21	19.51	17.92	-	24.28	Delta
S90	2021-08-09	Nasopharyngeal	31.39	31.89	31.21	Single_low	32.03	-	-	-	24.04	34.43	33.08	-	24.24	Delta
S91	2021-08-09	Nasopharyngeal	24.84	25.49	24.89	Single_low	24.46	-	-	-	24.26	27.57	25.61	-	24.34	Delta
S92	2021-08-11	Nasopharyngeal	22.45	24.13	22.28	Single_low	22.01	-	-	-	20.86	26.14	23.78	-	21.27	Delta
S93	2021-08-12	Nasopharyngeal	18.06	18.89	18.26	Double	20.62	-	-	-	25.55	23.75	22.04	-	26	Delta
S94	2021-08-25	Nasopharyngeal	13.65	14.35	13.64	Double	16.08	-	-	-	25.91	18.88	17.5	-	26.37	Delta

Single_high : Single with high amplification curve in RdRp/S

Single_low : Single with low amplification curve in RdRp/S
